# Molecular Cytogenetic Analysis of the Introgression between *Agropyron cristatum* P Genome and Wheat Genome

**DOI:** 10.3390/ijms222011208

**Published:** 2021-10-18

**Authors:** Zhi Zhang, Shenghui Zhou, Weihua Liu, Liqiang Song, Jinpeng Zhang, Haiming Han, Xinming Yang, Yida Lin, Xiuquan Li, Lihui Li

**Affiliations:** 1 National Key Facility for Crop Gene Resources and Genetic Improvement, Institute of Crop Sciences, Chinese Academy of Agricultural Sciences, Beijing 100081, China; zhangzhihkd@126.com (Z.Z.); zhoushenghui@caas.cn (S.Z.); liuweihua@caas.cn (W.L.); zhangjinpeng@caas.cn (J.Z.); hanhaiming@caas.cn (H.H.); yangxinming@caas.cn (X.Y.); linyida12138@163.com (Y.L.); lixiuquan@caas.cn (X.L.); 2 Center for Agricultural Resources Research, Institute of Genetics and Developmental Biology of Sciences, Shijiazhuang 050022, China; songliqiang.1988@163.com

**Keywords:** common wheat, *Agropyron cristatum*, translocation line, introgression, stripe rust

## Abstract

*Agropyron cristatum* (2*n* = 4*x* = 28, PPPP) is an important wild relative of common wheat (*Triticum aestivum* L., 2*n* = 6*x* = 42). A previous report showed that the wheat-*A. cristatum* 6P translocation line WAT655 carrying *A. cristatum* 6PS (0.81–1.00) exhibited high resistance to prevalent physiological races of stripe rust (CYR32 and CYR33). In this study, three disease resistance-related transcripts, which were mapped to *A. cristatum* 6PS (0.81–1.00) through the analysis of specific molecular markers, were acquired from among *A. cristatum* full-length transcripts. The BC_5_F_2_ and BC_5_F_2:3_ genetic populations of the translocation line WAT655 were analyzed by using three disease resistance-related gene markers, *A. cristatum* P genome-specific markers, and fluorescence in situ hybridization (FISH). The results revealed that the introgression between *A. cristatum* P genome and wheat genome was observed in progenies of the genetic populations of the translocation line WAT655 and the physical positions of the three genes were considerably adjacent on *A. cristatum* 6PS (0.81–1.00) according to the FISH results. Additionally, kompetitive allele-specific PCR (KASP) markers of the three genes were developed to detect and acquire 24 breeding lines selected from the progenies of the distant hybridization of wheat and *A. cristatum*, which showed resistance to physiological races of stripe rust (CYR32 and CYR33) and other desirable agronomic traits according to the field investigation. In conclusion, this study not only provides new insights into the introgression between *A. cristatum* P genome and wheat genome but also provides the desirable germplasms for breeding practice.

## 1. Introduction

Common wheat (*Triticum aestivum* L., 2*n* = 6*x* = 42, AABBDD) is one of the most cultivated cereal crops in the world. The total global wheat acreage was approximately 220 million hectares, and the global yield was more than 770 million tons in 2020 [1]. Wheat is a staple food crop that can provide starch, protein, vitamins, dietary fiber, and phytochemicals for humans, and wheat also provides approximately 20% of the calories consumed by humans [2]. However, wheat often suffers from various biotic and abiotic stresses during production, and the narrow genetic basis of wheat restricts its genetic improvement [3]. Wheat stripe rust is a fungal disease caused by the fungus *Puccinia striiformis* f. sp. *tritici* (*Pst*). Approximately 90% of cultivated wheat is susceptible to *Pst*, and more than five million tons of the wheat harvest are lost annually [4,5]. With the emergence of new *Pst* races and the variations in existing *Pst* races, many wheat varieties have lost resistance to stripe rust [6,7]. Therefore, providing new wheat germplasms with resistance to *Pst* infection is necessary for advancing wheat breeding.

Wild relatives of wheat are crucial gene resources for broadening the genetic basis of wheat and facilitating wheat breeding. Wild relatives of wheat carry numerous disease resistance genes [8,9]. Moreover, with the increasing number of successful distant hybridizations, an increasing number of exogenous genes from wild relatives, particularly disease resistance genes, have been transferred into common wheat. The powdery mildew resistance gene *Pm21* from *Haynaldia* *villosa* has been transferred into common wheat and widely applied in breeding practice [10,11,12]. Multiple stem rust resistance genes from *Triticum monococcum*, such as *Sr21*, *Sr22*, *Sr35*, and *Sr60*, have been cloned in common wheat [13,14,15,16,17]. Stripe rust resistance genes, such as *YrAS2388*, *Yr28*, *YrY201* from *Aegilops tauschii* [18,19,20,21,22], and *Yr15* from wild emmer wheat [23], have been cloned from common wheat. Hence, wild relatives of wheat are an abundant gene pool for providing disease resistance genes.

*Agropyron cristatum* (2*n* = 4*x* = 28, PPPP), an important wild relative of wheat, carries several desirable agronomic traits that can be applied in wheat breeding. With the achievement of the distant hybridization of wheat and *A. cristatum*, a series of wheat-*A. cristatum* derived lines were generated [24,25,26,27,28,29,30]. Many desirable genes from *A. cristatum* have been transferred into common wheat in the form of the translocation line. For example, *A. cristatum* P chromosomal segments carrying the powdery mildew resistance gene, the high-thousand grain weight (TGW) gene, and the high-grain number per spike (GNS) gene have been translocated into wheat chromosomes [28,29,31]. According to recent reports, the leaf rust resistance gene and the powdery mildew resistance gene from the *A. cristatum* 2P chromosome have been localized on the same segment of *A. cristatum* 2PL (0.66–0.86) [32], and both the stripe rust resistance gene and the leaf rust resistance gene were mapped to *A. cristatum* 6PS (0.81–1.00) [33]. Although wheat–*A. cristatum* translocation lines carry many desirable genes, they remain difficult to apply in wheat breeding because of the genetic drag caused by the exogenous chromosomal segments. Therefore, the identification of the introgression lines carrying smaller exogenous components is necessary for improving wheat breeding.

At present, over 80 wheat stripe rust resistance genes (*Yr1*–*Yr81*) have been named [34], however, a small number of stripe rust resistance (*Yr*) genes have been cloned. Of the *Yr* genes cloned so far, *Yr5*, *Yr7*, *YrSP*, and *YrAS2388R* encode nucleotide-binding domains and leucine-rich repeat domains [35,36]; and *Yr36* and *Yr15* encode kinase domains [23,37]. Moreover, in addition to stripe rust resistance genes, most of the other disease resistance genes in wheat also encode nucleotide-binding domains, leucine-rich repeat domains, or kinase domains [38]. For example, powdery mildew resistance genes (*Stpk*-*V*, *Pm21*, *Pm41*, and *Pm60*) [10,12,39,40] and stem rust resistance genes (*Sr22*, *Sr35*, *Sr45*, and *Sr60*) [15,16,17] encode nucleotide-binding domains, leucine-rich repeat domains, or kinase domains. Therefore, nucleotide-binding domains, leucine-rich repeat domains, and kinase domains can be considered as typical domains to help the clone of disease resistance genes.

In this study, to explore the introgression between *A. cristatum* P genome and wheat genome and detect *A. cristatum* P genomic components of breeding lines, the specific molecular markers and fluorescence in situ hybridization (FISH)/genomic in situ hybridization (GISH) probes made from the DNA of three bacterial artificial chromosome (BAC) clones, three disease resistance-related *A. cristatum* genes, and *A. cristatum* “Z559” were used to analyze the wheat–*A. cristatum* 6P derived lines. The introgression between *A. cristatum* P genome and wheat genome was observed, and the new breeding lines carrying *A. cristatum* P genomic components were acquired.

## 2. Results

### 2.1. The Acquisition of Disease Resistance-Related A. cristatum Genes of the Translocation Line WAT655

**Figure 1:**
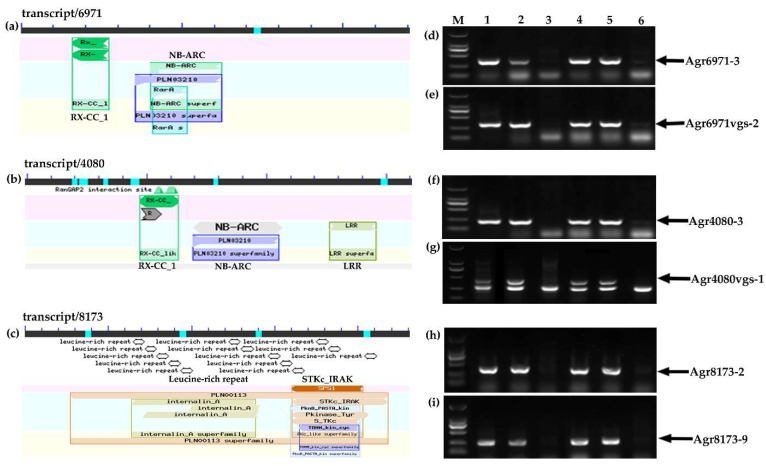
The functional annotations and the PCR amplification patterns of the molecular markers of three *A. cristatum* full-length transcripts. (**a**–**c**) The functional annotations of three full-length transcripts. (**d**–**i**) The gene-specific molecular markers of three full-length transcripts were used to trace *A. cristatum* 6PS (0.81–1.00). Lanes: M, DL2000 DNA marker; 1, Z559; 2, 4844-12; 3, Fukuho; 4, WAT655; 5, WAT638a; 6, WAT638b.

In previous reports, the wheat–*A. cristatum* 6P translocation line WAT655 carrying *A. cristatum* 6PS (0.81–1.00) exhibited high resistance to stripe rust (CYR32 and CYR33) and leaf rust in the adult stage [33,41]. To further explore the disease resistance genes of *A. cristatum* 6PS (0.81–1.00), the software TransDecoder (version v5.5.0) [42] was used to determine protein sequences of the *A. cristatum* full-length transcripts [43] and the software eggNOG-mapper [44] and BLAST from the NCBI website were used for the functional annotation. According to the information of the functional annotation, the disease resistance-related transcripts were searched from among *A. cristatum* full-length transcripts. Then, based on the sequences of disease resistance-related transcripts, molecular markers were developed to trace the *A. cristatum* 6P chromosomal segments of the translocation line WAT655. As a result, the specific molecular markers of three disease resistance-related transcripts can be mapped to *A. cristatum* 6PS (0.81–1.00) of the translocation line WAT655 (Figure 1 and Table 1).

### 2.2. The Preliminary Analysis of the Introgression between A. cristatum P Genome and Wheat Genome in the BC_5_F_2_ and BC_5_F_2:3_ Genetic Populations of the Translocation Line WAT655 Using the Molecular Markers

**Figure 2:**
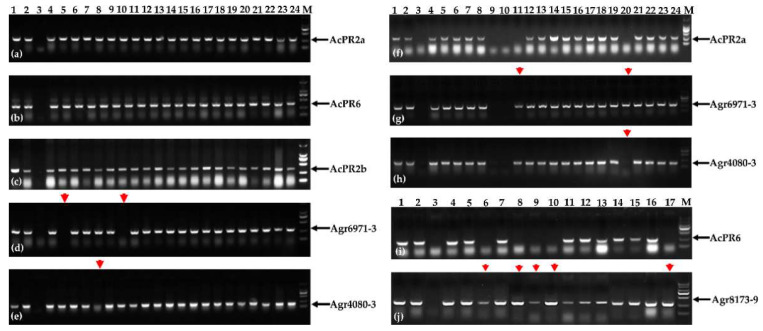
Then PCR amplification patterns of *A. cristatum* P genome-specific markers (AcPR2a, AcPR6, and AcPR2b) and gene makers (Agr6971-3, Agr4080-3, and Agr8173-9) in the genetic populations (BC_5_F_2_ and BC_5_F_2:3)_. (**a**–**e**) The detection results for the BC_5_F_2_ population of the translocation line WAT655. (**f**–**j**) The detection results for the BC_5_F_2:3_ population of the translocation line WAT655. The red arrows indicated the differences between P genome-specific markers and the gene makers in these plants; the black arrows indicated the diagnostic bands. Lanes: M, DL2000 DNA marker; 1, Z559; 2, 4844-12; 3, Fukuho; 4–24 of (**a**–**e**) the partial plants of the BC_5_F_2_ population; 4–24 of (**f**–**h**) the partial plants of the WAT655 BC_5_F_2:3_ population; 4–17 of (**i**,**j**) the partial plants of the BC_5_F_2:3_ population.

To trace *A. cristatum* 6P chromosomal segments in the BC_5_F_2_ population (2019–2020) of the translocation line WAT655, *A. cristatum* P genome-specific markers [45] (Table S1) and the gene markers from three disease resistance-related transcripts were utilized in this study. The detection results of the gene markers were different from the results of the *A. cristatum* P genome-specific markers in some plants of the BC_5_F_2_ population of the translocation line WAT655 (Figure 2). Preliminarily speculating, the introgression between *A. cristatum* 6PS (0.81–1.00) chromosomal segments and wheat chromosomes could occur in progenies of the BC_5_F_2_ population of the translocation line WAT655, which resulted in the differences between the two kinds of molecular markers in some plants.

To further confirm whether the introgression between *A. cristatum* 6PS (0.81–1.00) chromosomal segments and wheat chromosomes can occur in the common wheat background, the progenies from the BC_5_F_2_ population (2019–2020) of the translocation line WAT655 were planted in 2020–2021. The two kinds of molecular markers were utilized to detect P genomic components in the BC_5_F_2:3_ population, and the results revealed that some plants still showed differences between the gene markers and P genome-specific markers (Figure 2). These results further confirmed that the introgression of *A. cristatum* P genome and wheat genome can occur in the translocation line WAT655.

### 2.3. The Identification of the Introgression between A. cristatum P Genome and Wheat Genome in the Translocation Line WAT655

To acquire more sequence information of three disease resistance-related transcripts, the gene markers from the three transcripts were used to search *A. cristatum* “Z559” P genomic BAC bank, moreover, three BAC clones (BAC1013, BAC700, and BAC940) of three genes were acquired (Table 1). Pacific Biosciences technology was used to sequence the three BAC clones, and the software Hifiasm [46] was used to assemble the sequence data. This work was performed by BioMarker Company (Beijing, China).

**Figure 3:**
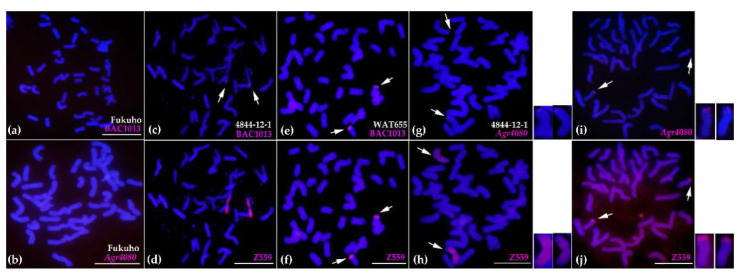
The FISH and GISH patterns of common wheat “Fukuho”, the substitution line 4844-12-1, and the translocation line WAT655 using the DNA of the BAC clone “BAC1013”, the gene *Agr4080*, and *A. cristatum* “Z559” as the probes. (**a**,**b**) The FISH patterns of common wheat “Fukuho” using the probes BAC1013 and *Agr4080*. (**c**,**e**) The FISH patterns of the substitution line 4844-12-1 and the translocation line WAT655 using the probe BAC1013. (**g**,**i**) The FISH patterns of the substitution line 4844-12-1 and the translocation line WAT655 using the probe *Agr4080*. (**d**–**j**) The GISH patterns of the substitution line 4844-12-1 and the translocation line WAT655 using the DNA of *A. cristatum* “Z559” as the probe. *A. cristatum* 6P chromosomal segments were in red, while wheat chromosomes were in blue strained by 4′,6-diamidino-2-phenylindole (DAPI). The white arrows indicated the *A. cristatum* chromatin in the wheat background. (scale bar = 10 µm).

**Figure 4:**
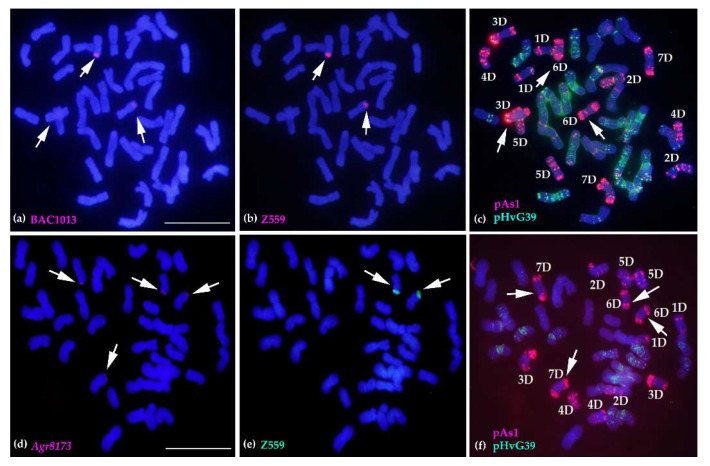
The cytogenetic analysis of the introgression in the translocation line WAT655 using the DNA of *A. cristatum* BAC clone BAC1013, the gene *Agr8173*, *A. cristatum* “Z559”, pAs1, and pHvG39 as the probes. (**a**,**d**) The probes BAC1013 (red) and *Agr8173* (red) were used for the first round of FISH, respectively. The white arrows indicated the *A. cristatum* chromatin in the wheat background. (**b**,**e**) The DNA of *A. cristatum* “Z559” as the probes (red/green) was used for the second round of GISH on the same slides. The white arrows indicated the *A. cristatum* chromatin in the wheat background. (**c**,**f**) The probes pAs1 (red) and pHvG39 (green) were used for the third round of FISH on the same slides. The white arrows indicated the wheat chromosomes that the *A. cristatum* chromatins were located on. Wheat chromosomes were in blue strained by DAPI. (scale bar = 10 µm).

**Figure 5:**
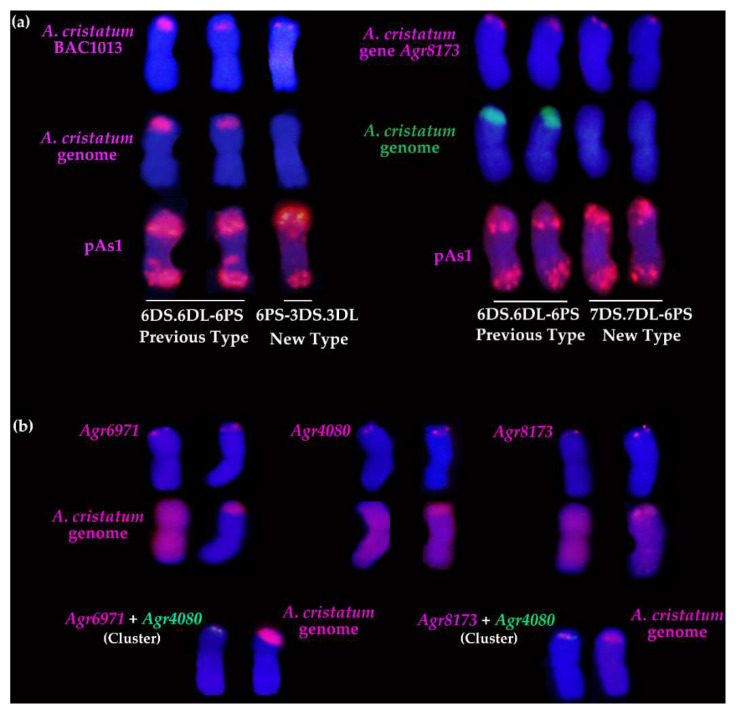
The production of the new types through the introgression and the physical positions of the three genes on *A. cristatum* 6PS (0.81–1.00) in the translocation line WAT655. (**a**) The new translocated types were produced through the introgression between *A. cristatum* P genome and wheat genome. The probes BAC1013, *Agr8173*, and pAs1 were in red; the probes made from *A. cristatum* P genomic DNA were in red or green. (**b**) The physical positions of the three genes *Agr6971*, *Agr4080*, and *Agr8173* on *A. cristatum* 6PS (0.81–1.00). The probes *Agr6971*, *Agr8173*, and the probe made from the DNA of *A. cristatum* “Z559” were in red, while the probe *Agr4080* was in green. The FISH/GISH patterns of chromosomes in (**a**,**b**) are from Figure 4 and Figures S2–S4.

To further analyze the introgression between *A. cristatum* P genome and wheat genome, the DNA of the three BAC clones, three disease resistance-related genes, and *A. cristatum* “Z559” was utilized as the probes to perform FISH and GISH in common wheat “Fukuho”, the translocation line WAT655, and the substitution line 4844-12-1 (Figure 3 and Figure 4, Figures S1–S3). The FISH and GISH probes could not hybridize with the chromosomal DNA of common wheat “Fukuho” (Figure 3 and Figure S2). The signals of the FISH probes made from the DNA of the BAC clones (BAC1013, BAC700, and BAC940) and three genes (*Agr6971*, *Agr4080*, and *Agr8173*) were mapped to *A. cristatum* 6PS (0.81–1.00) of the translocation line WAT655 and the *A. cristatum* 6PS terminal of the substitution line 4844-12-1 (Figure 3, Figures S1–S3). Significantly, the FISH probe BAC1013 traced one *A. cristatum* 6PS chromosomal segment on the wheat chromosome 3DS in addition to the previously translocated wheat chromosome 6D (Figure 4 and Figure 5); moreover, the probe *Agr8173* detected *A. cristatum* 6PS chromosomal segments on the wheat chromosome 7DL (Figure 4 and Figure 5). Therefore, the above results indicated that the introgression between *A. cristatum* P genomic chromosomal segments and wheat chromosomes can occur in progenies of the genetic population of the translocation line WAT655.

### 2.4. Molecular Cytogenetic Analysis of the Physical Positions of Three Disease Resistance-Related Genes

To explore the physical positional relationships of the three genes on *A. cristatum* 6PS (0.81–1.00) of the translocation line WAT655, the DNA of *Agr6971* and *Agr8173* was labeled with Texas Red^®^-5-dCTP (red), and the DNA of *Agr4080* was labeled with Fluorescein-12-dUTP (green). The probes *Agr6971* (red) and *Agr4080* (green) were simultaneously used for one FISH experiment (Figure 5 and Figure S4), and the probes *Agr8173* (red) and *Agr4080* (green) were simultaneously used for another (Figure 5 and Figure S4). The signals of the FISH probes showed that *Agr6971* (red) and *Agr4080* (green) were localized at one overlapping zone of *A. cristatum* 6PS (0.81–1.00); moreover, *Agr8173* (red) and *Agr4080* (green) were also localized at one overlapping zone of *A. cristatum* 6PS (0.81–1.00). These FISH results revealed that three genes were localized at the considerably adjacent positions on *A. cristatum* 6PS (0.81–1.00) (Figure 5), which could constitute a potential disease resistance-related gene cluster.

### 2.5. Tracing A. cristatum P Genomic Components of Breeding Lines Selected from Progenies of Distant Hybridization between A. cristatum and Wheat

**Figure 6:**
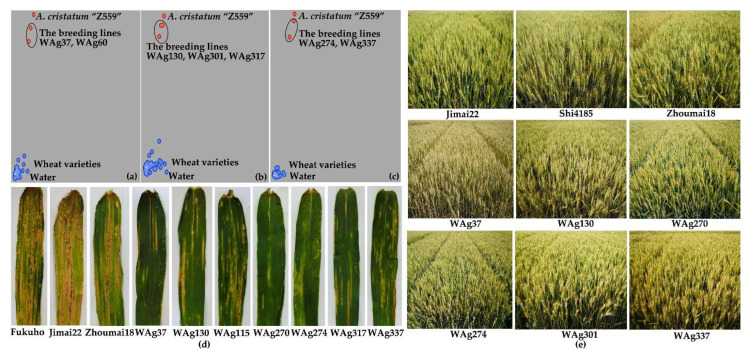
The detection of *A. cristatum* P genomic components using KASP markers and the investigation of agronomic traits of breeding lines. (**a**–**c**) The detection results of Agr6971kps-4, Agr4080kps-2, and Agr4080kps-4, respectively. The genotype of *A. cristatum* “Z559” was in red; the genotype of wheat was in blue. The genotype of the breeding lines was in the circles. (**d**,**e**) The agronomic traits of partial breeding lines including resistance to stripe rust and other agronomic traits in the field.

**Figure 7:**
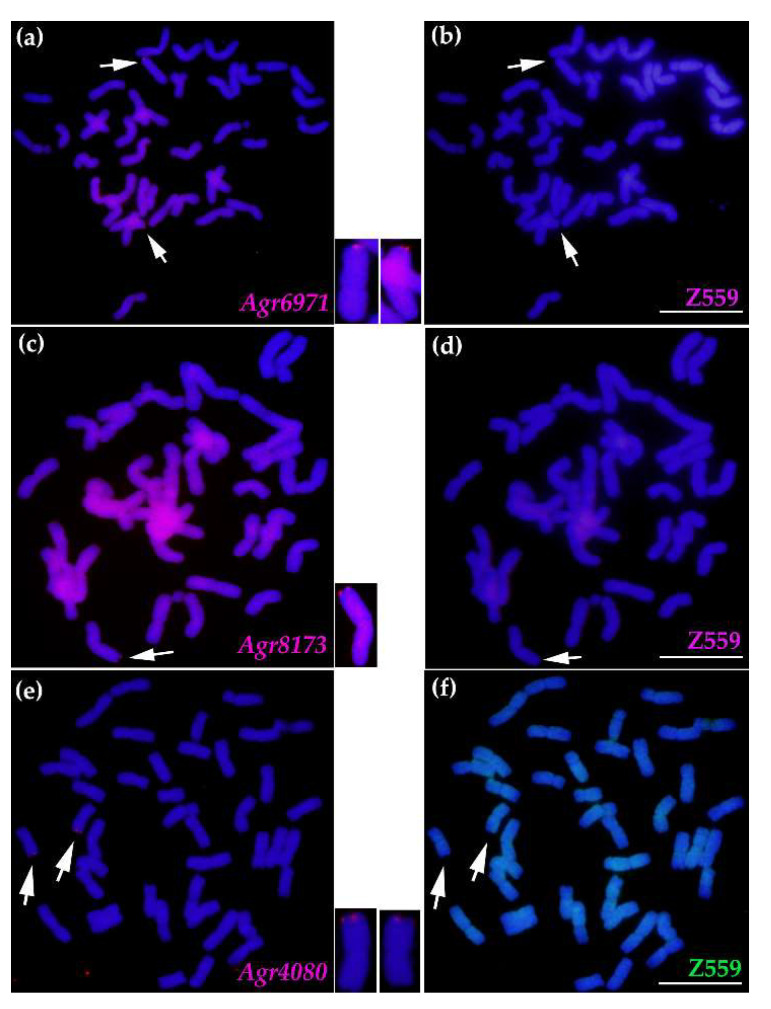
The FISH detection of *A. cristatum* P genomic components of the partial breeding lines using three gene FISH probes. (**a**,**c**,**e**) The *A. cristatum* P genomic components of the breeding lines can be detected using three gene probes. *A. cristatum* 6P chromosomal segments were in red, while wheat chromosomes were in blue strained by DAPI. The white arrows indicated the *A. cristatum* chromatin in the wheat background. (**b**,**d**,**f**) The *A. cristatum* P genomic components of the breeding lines cannot be traced by the DNA of *A. cristatum* “Z559” as the probe. (scale bar = 10 µm).

To trace the *A. cristatum* P genomic components of breeding lines selected from progenies of distant hybridization between *A. cristatum* and wheat, the KASP molecular markers of the three genes were developed according to the results of the alignment between the sequences of three genes and the Chinese Spring genome sequences RefSeq v1.0 [47] (Figure S5 and Table S2). The KASP molecular markers were used to trace the *A. cristatum* 6P components of the BC_5_F_2:3_ population of the translocation line WAT655 and the *A. cristatum* 6P components of breeding lines, and the results were completely coincident with those of standard PCR detection of the gene markers relying on agarose gel electrophoresis (Figure S6). Meanwhile, 24 breeding lines carrying *A. cristatum* P genomic components were acquired from among 500 breeding lines (Figure 6 and Table 2). The three gene FISH probes were used to detect the *A. cristatum* P genomic components of the part of 24 breeding lines, and the breeding lines showed the *A. cristatum* P genomic components can be detected through the FISH probes (Figure 7). Additionally, these breeding lines exhibited resistance or moderate resistance to stripe rust (CYR32 and CYR33) according to the investigation in the field (Figure 6 and Table 2). These breeding lines also exhibited other desirable agronomic traits in the field (Figure 6 and Table 2). Therefore, *A. cristatum* P genomic components of these breeding lines can be traced by the molecular markers and the FISH probes, and these breeding lines can also promote wheat breeding.

## 3. Discussion

Distant hybridization is an efficient and important method and contributes much to wheat breeding for disease resistance. For example, the powdery mildew gene *Pm21* of *Haynaldia* *villosa* 6VS has been widely applied in wheat breeding [11,12]; the 1BL•1RS translocation line carries many disease resistance genes, such as the stripe rust resistance gene *Yr9*, the powdery mildew resistance gene *Pm8*, the stem rust resistance gene *Sr31*, and the leaf rust resistance gene *Lr26* [3,48,49,50]. *A. cristatum* is one of the most important wild relatives of common wheat and carries many desirable agronomic traits [8,9]. In recent years, many desirable genes from *A. cristatum* have been transferred into common wheat, such as the stripe rust resistance, the leaf rust resistance, the powdery mildew, the high-GNS, and the high-TGW genes [28,29,31,32,33,41,51,52,53,54]. These genes from *A. cristatum* are usually mapped on relatively large *A. cristatum* chromosomal segments of translocation lines. However, translocation lines are not easy to apply in breeding practice because of the genetic drag caused by the translocated segments, so many breeders desire the introgression lines carrying smaller exogenous genetic components for breeding. In this study, gene markers, *A. cristatum* genome-specific markers, and FISH/GISH probes were used to analyze the wheat–*A. cristatum* 6P translocation line WAT655. The evidence of the introgression between *A. cristatum* 6P chromosomal segments and wheat chromosomes was observed (Figure 4, Figure 5, Figure 6 and Figure 7), and these three genes could be mapped to the considerably adjacent physical positions on *A. cristatum* 6PS (0.81–1.00) according to the FISH signals (Figure 5). Additionally, 24 breeding lines carrying *A. cristatum* P genomic components were acquired through molecular cytogenetic analysis (Figure 6 and Figure 7). These breeding lines exhibited favorable breeding agronomic traits including resistance to stripe rust (CYR32 and CYR33) according to the investigation in the field, which also indicated that three disease resistance-related genes were closely associated with the resistance to stripe rust. Moreover, the molecular markers designed from the sequences of three disease resistance-related genes can also promote the application of marker-assisted selection in distant hybridization of wheat and *A. cristatum* and wheat breeding. In addition, to further verify the function of three disease resistance-related genes, transgenic work involving coding sequences (CDSs) and full-length sequences of three disease resistance-related genes is already underway. Therefore, this study not only provides new insights into the introgression between *A. cristatum* P genome and wheat genome but also provides a new approach for acquiring valuable breeding lines.

Some disease resistance genes have been shown to be distributed in the form of a gene cluster. For example, the powdery mildew resistance genes *Pm21* and *Stpk*-*V* were mapped to the same locus of *Haynaldia* *villosa* 6VS bin (FL 0.45–0.58) [11,12]; the stripe rust resistance genes *Yr5*, *Yr7*, and *YrSP* were on a gene cluster [35]. Both the powdery mildew resistance gene and the leaf rust resistance gene of *A. cristatum* 2P chromosome were mapped to *A. cristatum* 2PL (0.66–0.86) [29,32]. The stripe rust resistance gene and the leaf rust resistance gene were localized on *A. cristatum* 6PS (0.81–1.00) of the translocation line WAT655 [33,41,49]. In this study, the DNA of three disease resistance-related genes was used as FISH probes to identify the physical positions of the genes on *A. cristatum* 6PS (0.81–1.00). The results revealed that the FISH signals of the probes were located at the overlapping positions of *A. cristatum* 6PS (0.81–1.00) (Figure 5 and Figure S4), therefore, the three genes could be mapped to the considerably adjacent physical positions, which could constitute a potential disease resistance-related gene cluster on *A. cristatum* 6PS (0.81–1.00).

*A. cristatum* P genome and wheat genome were revealed to present a homoeologous relationship by using a wheat 660K SNP array, and apparent chromosomal rearrangements and introgression spread throughout the P genome were observed [55]. In addition, genetic rearrangements of *A. cristatum* 6P chromosomes could usually occur at the terminal of chromosomes by analyzing the different wheat–*A. cristatum* 6P addition lines [56]. In this study, the results of FISH using the DNA of the BAC clones and three disease resistance-related genes as probes indicated that the introgression can occur between *A. cristatum* 6P chromosomal segments and wheat chromosomes (Figure 4 and Figure 5), moreover, the observed introgression also occurred in the terminal of the chromosomes (Figure 4 and Figure 5). The mechanism of the introgression could involve chromosomal rearrangements between *A. cristatum* P genome and wheat genome resulting from their homoeologous relationship.

## 4. Materials and Methods

### 4.1. Plant Materials

The plant materials utilized in this study included the following: *A. cristatum* accession “Z559” (2*n* = 4*x* = 28, PPPP, from Xinjiang, China), a wheat–*A. cristatum* 6P disomic addition line 4844-12 (2*n* = 44) [27], a wheat-*A. cristatum* 6P disomic substitution line 4844-12-1 (2*n* = 42) (Table 3 and Figure S7), a wheat–*A. cristatum* 6P terminal translocation line WAT655 (Figure S8), a 6PS whole-arm translocation line WAT638a, a 6PL whole-arm translocation line WAT638b [33,41] (Table 3), *Triticum aestivum* cv. Fukuhokomugi (Fukuho) (2*n* = 6*x* = 42, AABBDD), wheat varieties (Zhoumai18, Jimai22, Shi4185, Luyuan502, Gaocheng8901, and Xinong979), and 500 breeding lines (WAg1–WAg500) selected from the progenies of the distant hybridization of *A. cristatum* and wheat. The wheat–*A. cristatum* 6P disomic addition line 4844-12 was produced via distant hybridization of *A. cristatum* accession “Z559” and common wheat “Fukuho” [27]. The wheat–*A. cristatum* 6P disomic substitution line 4844-12-1 was produced from the progenies of wheat–*A. cristatum* 6P disomic addition line 4844-12. Wheat–*A. cristatum* 6P translocation lines WAT655, WAT638a, and WAT638b were produced by radiating hybrid plants of the wheat–*A. cristatum* 6P disomic addition line 4844-12, which were acquired via strict backcrossing with common wheat “Fukuho” as the recurrent parent and self-pollination. The BC_5_F_2_ and BC_5_F_2:3_ genetic populations of the translocation line WAT655 were acquired via five-time strict backcrossing (BC_5_) with common wheat “Fukuho” as the recurrent parent and strict self-pollination. A total of 500 breeding lines were selected from the progenies of the distant hybridization of *A. cristatum* and wheat. All of the plant materials were preserved at the Center of Crop Germplasm Resources Research at the Institute of Crop Science, Chinese Academy of Agricultural Sciences (Beijing, China).

### 4.2. Molecular Cytogenetic Analysis

*A. cristatum* P genome-specific markers were designed from P genome-specific repeat sequences (Table S1) [42]. *A. cristatum* P genome-specific markers were also used to detect *A. cristatum* 6P chromosomal segments in the genetic populations of the translocation line WAT655. The *A. cristatum* full-length transcripts were analyzed by using TransDecoder (version v5.5.0) [43] to determine the CDSs and protein sequences, and the software eggNOG-mapper [44] and BLAST from the National Center of Biotechnology Information (NCBI) website were used to annotate these sequences. The disease resistance-related genes were searched, and specific molecular markers were developed for the analysis of wheat–*A. cristatum* 6P translocation line WAT655. Additionally, the specific sequences of three genes were used to design KASP markers to trace *A. cristatum* P genomic components in wheat–*A. cristatum* breeding lines (Figure S5 and Table S2). HiGeno 2x Probe Mix (JasonGen, Beijing, China) was used for the KASP reaction, and PCR products were detected with a PHERAstarplus SNP genotyping instrument (LGC Science Shanghai Ltd., Shanghai, China). The software KlusterCallerTM was used for genotyping assays.

FISH and GISH were performed in root tip cells, as described by Cuadrado et al. [57]. The DNA of three BAC clones, three disease resistance-related genes, and *A. cristatum* “Z559” were used as FISH/GISH probes, and the genomic DNA of common wheat “Fukuho” was used as the blocker. The FISH/GISH probes and the blocker were used at a 1:40 ratio. Oligo-pTa535-1 (red) and Oligo-pSc119.2-1 (green) [58] (Table S1) were used to analyze the wheat chromosomes in the substitution line 4844-12-1. The QIAGEN Large-Construct Kit (Qiagen, Germany) was used to extract the DNA of the three BAC clones. The DNA of three disease resistance-related genes was acquired from the three *A. cristatum* “Z559” genomic BAC clones, respectively. The DNA of *A. cristatum* “Z559”, the three BAC clones (BAC1013, BAC700, and BAC940), the three genes (*Agr6971*, *Agr4080*, and *Agr8173*), and pAs1 [59] were labeled with Texas Red^®^-5-dCTP (red) (PerkinElmer, Waltham Mass, MA, USA). The DNA of *A. cristatum* “Z559”, the gene *Agr4080*, and pHvG39 [59] was labeled with Fluorescein-12-dUTP (green) (PerkinElmer, Waltham Mass, MA, USA). VECTASHIELD^®^ Antifade Mounting Medium with DAPI (Vector Laboratories, Burlingame, CA, USA) was used for the FISH/GISH experiments. Signals were captured using an OLYMPUS AX80 (Olympus Corporation, Tokyo, Japan) fluorescence microscope with a CCD camera (Diagnostic Institute, Inc., Sterling Height, MI, USA) and processed with Photoshop CS 3.0.

### 4.3. Evaluation of the Agronomic Traits of Wheat–A. cristatum Breeding Lines in the Field

To evaluate stripe rust resistance, the wheat-*A. cristatum* 6P disomic addition line 4844-12, the wheat–*A. cristatum* 6P disomic substitution line 4844-12-1, the wheat-*A. cristatum* 6P translocation line WAT655, wheat varieties (Zhoumai18, Jimai22, Shi4185, Luyuan502, Gaocheng8901, and Xinong979), 500 breeding lines selected from the progenies of the distant hybridization of *A. cristatum* and wheat, and the common wheat cultivar “Fukuho” were planted in a randomized complete block design with three replicates in the field in Beijing (39°54′20″ N, 116°25′29″ E, China) during 2019–2020. Twenty grains of each material were planted in 2.0 m rows that were spaced 0.3 m apart. The common wheat “Fukuho” was used as the control. The prevalent physiological races of stripe rust CYR32 and CYR33 were used to inoculate the plant materials at the elongation stage in the field in Beijing. The infection type (IT) score ranged from 0 to 9 (0: no visible symptoms; 1–2: necrotic flecks or necrotic areas without sporulation; 3–4: trace or light sporulation; 5–6: intermediate or moderate sporulation; 7–8: abundant sporulation; 9: no necrosis or chlorosis and abundant sporulation) [60]. Plants with IT scores of 0–2 were considered as highly resistant type (HR); plants with IT scores of 3–4 were considered as moderately resistant type (MR); plants with IT scores of 5–6 were moderately susceptible type (MS); plants with IT scores of 5–9 were considered as highly susceptible type (HS).

To evaluate agronomic traits, wheat varieties (Zhoumai18, Jimai22, Shi4185, Luyuan502, Gaocheng8901, and Xinong979) and 500 breeding lines were planted in 1.2 × 6 m^2^ plots with four replicates in the field in Xinxiang (35°18′13″ N, 113°55′15″ E, Henan Province, China) during 2019–2020. The plant height, plot yield, and thousand grain weight of each material were measured. Statistical Analysis System (Version 9.4, SAS Institute, Cary, NC, USA) was used for statistical analysis in this study. Analysis of variance was performed to test the differences in the plant height, the plot yield, and the thousand grain weight among the breeding lines. The “mean ± SD” was used to represent the data.

## 5. Conclusions

In the previous report, the wheat–*A. cristatum* translocation line WAT655 carrying *A. cristatum* 6PS (0.81–1.00) exhibited the high resistance to prevalent physiological races of stripe rust (CYR32 and CYR33). In the present study, three disease resistance-related *A. cristatum* genes were acquired, which can be mapped to *A. cristatum* 6PS (0.81–1.00) carried by the translocation line WAT655. The three disease resistance-related genes could constitute a potential gene cluster through molecular cytogenetic analysis. Moreover, the introgression between *A. cristatum* P genome and wheat genome was observed in the translocation line WAT655 and the breeding lines carrying *A. cristatum* P genomic components were acquired by using the molecular markers and the FISH probes of the three genes. According to the resistance to stripe rust of the breeding lines, the three genes could be closely associated with the resistance to stripe rust. Therefore, this study provides new insights into the introgression between *A. cristatum* P genome and wheat genome and the desirable germplasms for breeding practice.

## Figures and Tables

**Table 1:** Sequences of molecular markers designed according to *A. cristatum* transcript sequences.

Transcript Id	Gene Name	BAC Clone	Primer Name	Left Primer (5′–3′)	Right Primer (5′–3′)	Annealing Temperature
transcript/6971	*Agr6971*	BAC1013	Agr6971-3	TCACCAAAGATCGAGCTCCT	ACCCGTCTGCAGATTGTACC	60 °C
			Agr6971vgs-2	GACCAGCTAGACAACCAGGT	TGCTGTTTGGGACCATCTCT	60 °C
transcript/4080	*Agr4080*	BAC700	Agr4080-3	GGGCGGTTTTACTTCACAAA	ACTTGCAGCTGTCAATGTGC	60 °C
			Agr4080-4	TGAAACTGGATGGACGATGA	GTGCTGCTGTGGTGTTGACT	60 °C
			Agr4080vgs-1	CCGAGGTGGCGAATGAACTTGT	AGAGATGGAGGCTGTGGTGACT	60 °C
			Agr4080cds-6	TTGTCGGACTAATAATAGCGC	TGCCGAGATAATGGGGTT	60 °C
transcript/8173	*Agr8173*	BAC940	Agr8173-2	GGTCCCATACCTCCCAGTTT	TCGATGAAAGGTCCAGTTCC	60 °C
			Agr8173-9	ATCGTTGGCCAATTCGATAG	CAGCAGAAGGAGCATGTTGA	60 °C
			Agr8173-6	TGATCATTGTGGAGACCGGA	CACCCTCTTTTGGCAACCTT	60 °C

PCR Program: 95 °C 5 mins; 95 °C 45 s, Annealing temperature °C 45 s, 72 °C 45 s, 35 cycles; 72 °C 5 min.

**Table 2:** The agronomic traits of wheat–*A. cristatum* breeding lines in 2019–2020.

Materials	Plant Height (cm)	Plot Yields (kg)	Thousand Grain Weight (g)	Stripe Rust Response
Jimai22	93.06 ± 1.41 ^a^	7.52 ± 0.24 ^c^	41.85 ± 0.28 ^c^	S
Zhoumai18	86.32 ± 2.15 ^c^	6.78 ± 0.26 ^e^	44.61 ± 0.42 ^b^	S
WAg20	77.25 ± 1.82 ^e^	6.09 ± 0.18 ^g^	\	R
WAg37	80.12 ± 2.65 ^d^	7.16 ± 0.38 ^d^	46.75 ± 0.45 ^a^	R
WAg38	80.50 ± 2.37 ^d^	6.59 ± 0.16 ^ef^	\	R
WAg60	90.61 ± 3.56 ^b^	8.40 ± 0.27 ^a^	43.10 ± 0.32 ^b^	MR
WAg115	83.21 ± 2.73 ^cd^	6.77 ± 0.23 ^e^	\	MR
WAg130	85.36 ± 4.01 ^c^	8.04 ± 0.26 ^b^	42.23 ± 0.24 ^bc^	MR
WAg165	80.58 ± 2.13 ^d^	7.49 ± 0.14 ^c^	\	R
WAg220	75.42 ± 2.87 ^e^	7.63 ± 0.20 ^c^	\	R
WAg270	76.09 ± 3.25 ^e^	7.35 ± 0.17 ^cd^	\	R
WAg274	82.20 ± 3.35 ^d^	6.46 ± 0.18 ^f^	\	R
WAg301	85.23 ± 3.89 ^c^	8.37 ± 0.26 ^a^	40.84 ± 0.25 ^c^	MR
WAg308	85.25 ± 2.10 ^c^	7.07 ± 0.35 ^d^	\	MR
WAg314	80.45 ± 2.34 ^d^	7.09 ± 0.19 ^d^	\	MR
WAg317	85.69 ± 3.14 ^c^	7.25 ± 0.15 ^d^	\	R
WAg322	87.57 ± 2.69 ^c^	8.32 ± 0.38 ^a^	39.73 ± 0.27 ^cd^	MR
WAg330	82.63 ± 1.82 ^d^	8.11 ± 0.20 ^ab^	37.89 ± 0.38 ^d^	MR
WAg335	80.33 ± 1.95 ^d^	7.52 ± 0.27 ^c^	38.96 ± 0.46 ^d^	R
WAg337	83.28 ± 2.74 ^cd^	8.22 ± 0.24 ^a^	41.08 ± 0.47 ^c^	MR
WAg339	80.29 ± 2.58 ^d^	7.13 ± 0.16 ^d^	38.66 ± 0.61 ^d^	R
WAg409	81.35 ± 3.46 ^d^	7.08 ± 0.13 ^d^	39.32 ± 0.39 ^d^	MR
WAg457	82.24 ± 2.09 ^d^	7.84 ± 0.23 ^b^	43.80 ± 0.46 ^b^	R
WAg466	75.49 ± 2.51 ^e^	7.45 ± 0.34 ^c^	38.58 ± 0.52 ^d^	MR
WAg475	85.41 ± 2.59 ^c^	8.24 ± 0.16 ^a^	45.94 ± 0.56 ^a^	R
WAg480	85.17 ± 2.49 ^c^	7.96 ± 0.30 ^b^	41.84 ± 0.23 ^c^	R

Significant differences in the mean ± standard deviation (SD) are indicated at the probability levels of *p* = 0.05 (lowercase letters), based on Duncan’s multiple range test (analysis of variance by SAS 9.4).

**Table 3:** The information of the plant materials.

Materials	Zygosity	Progeny	Type	6P Segment Size	Chromosome Constitution	Reference
4844-12-1	Homozygous		disomic substitution line	6P	42 (6P/6D)	
4844-12	Homozygous		disomic addition line	6P	44	Wu et al. [27]
WAT638b	Homozygous	BC_2_F_6_	6AS•6PL	6PL arm	42	Zhang et al. [33]; Song et al. [41]
WAT638a	Homozygous	BC_2_F_6_	6PS•6AL	6PS arm	42	
WAT655	Homozygous	BC_2_F_6_	6DS•6DL-6PS	6PS (0.81–1.00)	42	
	Heterozygous	BC_5_F_2_, BC_5_F_2:3_				

All of the plant materials were preserved at the Center of Crop Germplasm Resources Research at the Institute of Crop Science, Chinese Academy of Agricultural Sciences.

## Data Availability

The datasets of *Agropyron cristatum* BAC clones generated during and/or analyzed during the current study are not publicly available due to the transgenic work of the genes in progress but are available from the corresponding author on reasonable request.

## Supplementary Materials

The following are available online at https://www.mdpi.com/article/10.3390/ijms222011208/s1.

## Abbreviations

FISH	Fluorescence in situ hybridization
GISH	Genomic in situ hybridization
BAC	Bacterial artificial chromosome
KASP	Kompetitive allele-specific PCR
SD	Standard Deviation
*Pst*	*Puccinia striiformis* f. sp. *tritici*
TGW	Thousand grain weight
GNS	Grain number per spike
NCBI	National Center of Biotechnology Information
DAPI	4,6-diamino-2-phenyl indole
IT	Infection type
