# Supplementary material for: Molecular Cytogenetic Analysis of the Introgression between Agropyron cristatum P Genome and Wheat Genome
Source: Int J Mol Sci. 2021 Oct 18;22(20):11208. doi: 10.3390/ijms222011208 (PMC8539888; doi:10.3390/ijms222011208)
Supplement: Supplementary file 1 [file ijms-22-11208-s001.zip › Supplementary.pdf]

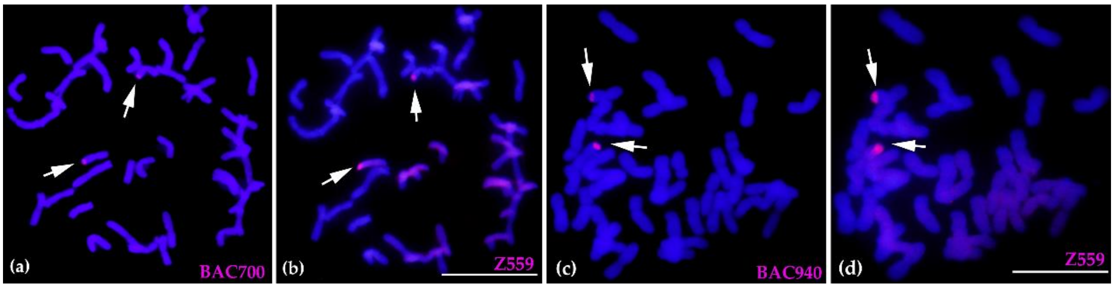


**Figure S1.** FISH and GISH patterns of the translocation line WAT655 using the DNA of *A. cristatum* BAC clones and *A. cristatum* “Z559” as the probes. (a) and (c) The FISH patterns from the probes BAC700 and BAC940, respectively; (b) and (d) The GISH patterns from *A. cristatum* “Z559” as the probes. *A. cristatum* 6P chromosomal segments were in red, while wheat chromosomes were in blue strained by DAPI. (scale bar=10 μm).

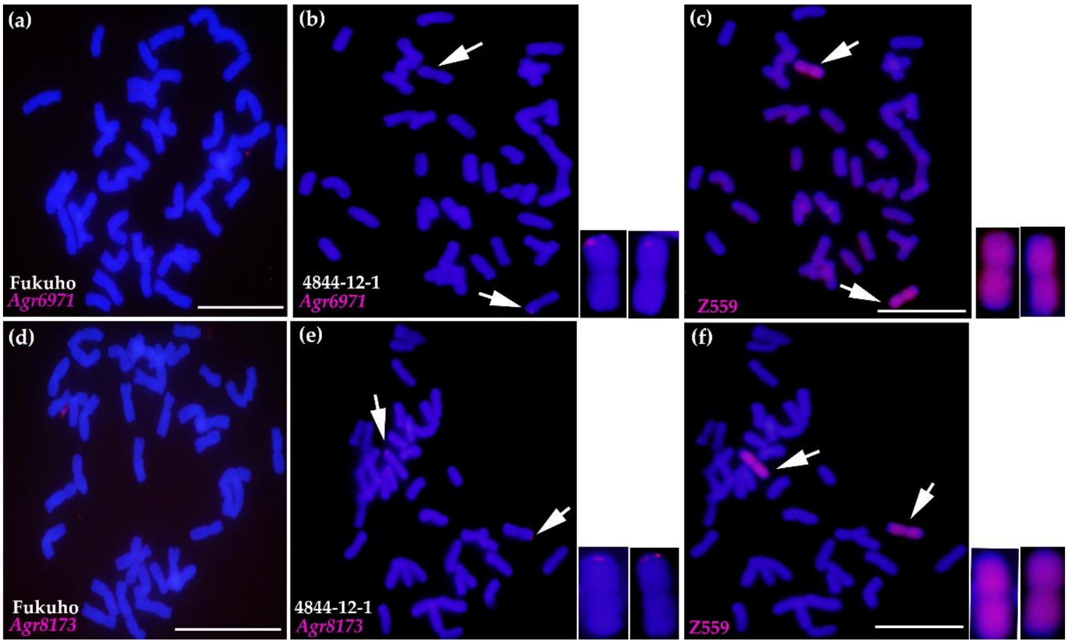


**Figure S2.** FISH and GISH patterns of the common wheat "Fukuho" and the substitution line 4844-12-1 using the DNA of the disease resistance-related genes and *A. cristatum* "Z559" as probes. (a) and (d) The probes *Agr6971* and *Agr8173* could not hybridize with chromosomal DNA of common wheat "Fukuho". (b) and (e) The DNA of the genes (*Agr6971* and *Agr8173*) was used as the probes for the first round of FISH in the substitution line 4844-12-1; (c) and (f) *A. cristatum* "Z559" DNA was used as the probe for the second round of GISH on the same slides in the substitution line 4844-12-1. *A. cristatum* 6P chromosomal segments were in red, while wheat chromosomes were in blue strained by DAPI. (scale bar = 10 μm).

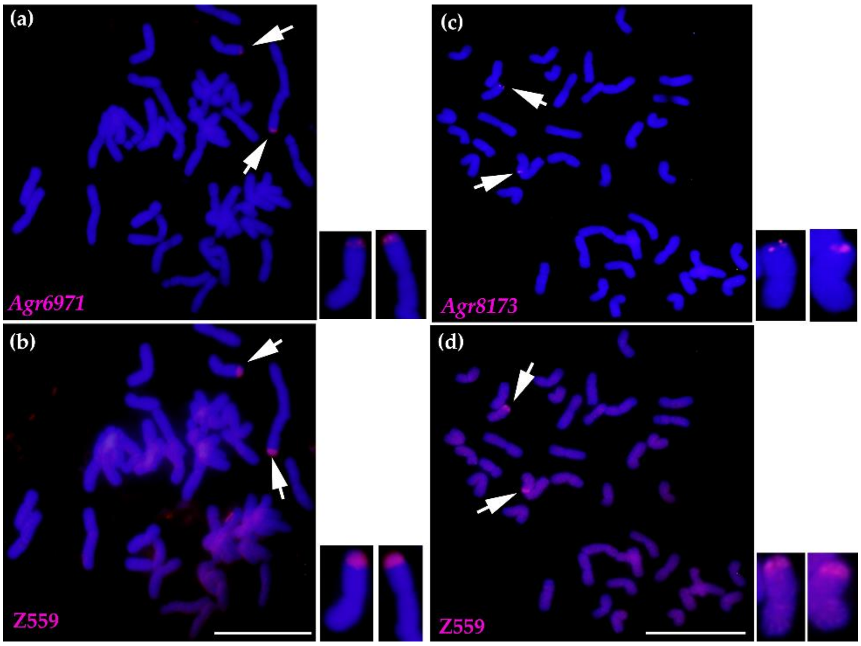


**Figure S3.** FISH and GISH patterns of the translocation line WAT655 using the DNA of the disease resistance-related genes and *A. cristatum* "Z559" as the probes. (a) and (c) The DNA of the genes (*Agr6971* and *Agr8173*) was used as the probes for the first round of FISH; (b) and (d) The DNA of *A. cristatum* "Z559" was used as the probes for the second round of GISH on the same slides. *A. cristatum* 6P chromosomal segments were in red, while wheat chromosomes were in blue strained by DAPI. (scale bar = 10 μm).

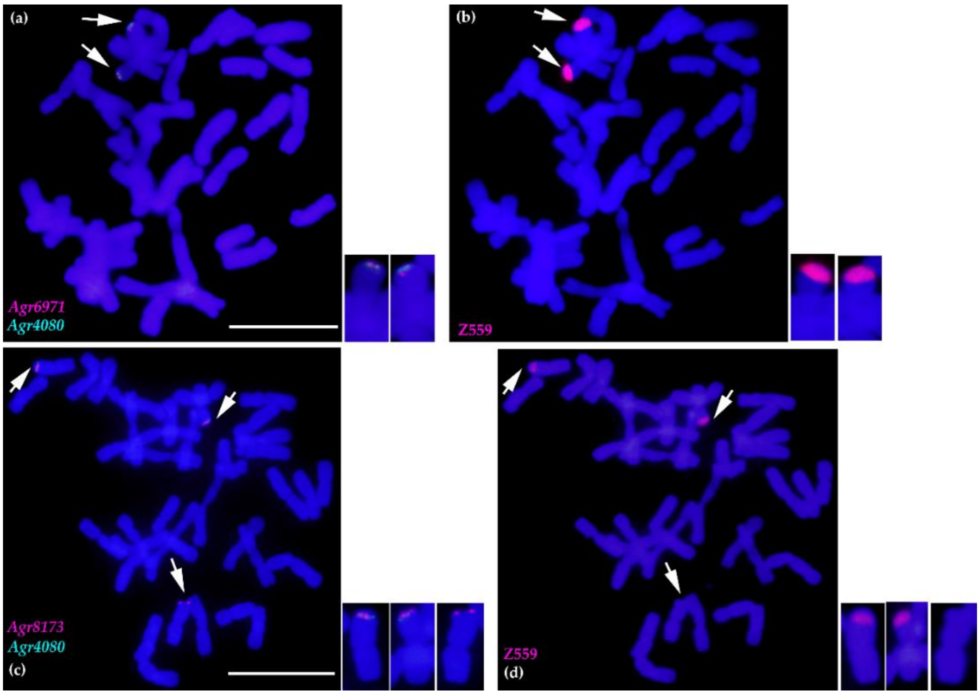


**Figure S4.** FISH and GISH patterns of the translocation line WAT655 using the DNA of three genes and *A. cristatum* "Z559" as the probes. (a) and (b) The signals of the probes *Agr6971* (red) and *Agr4080* (green) were simultaneously mapped to the overlapping zones of *A. cristatum* 6PS (0.81–1.00) of the translocation line WAT655, moreover, (c) and (d) The signals of the probes *Agr8173* (red) and *Agr4080* (green) also were mapped to the overlapping zones. (scale bar=10 μm).

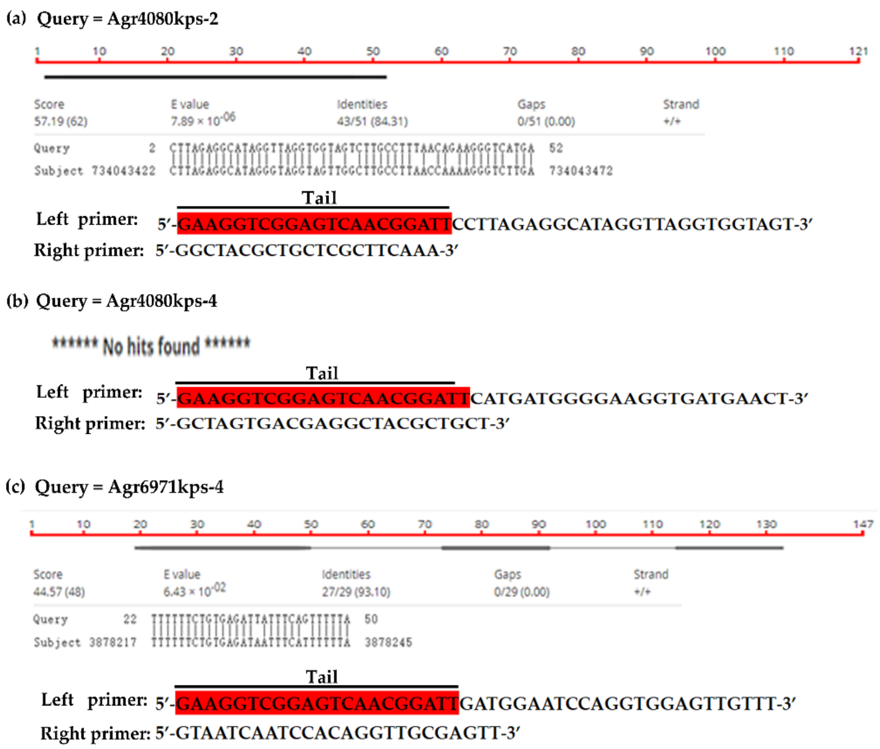


**Figure S5.** The alignment results of the sequences amplified by the KASP markers (Agr4080kps-2, Agr4080kps-4, and Agr6971kps-4) and Chinese Spring genome sequences RefSeq v1.0 and the sequences of the KAPS markers.

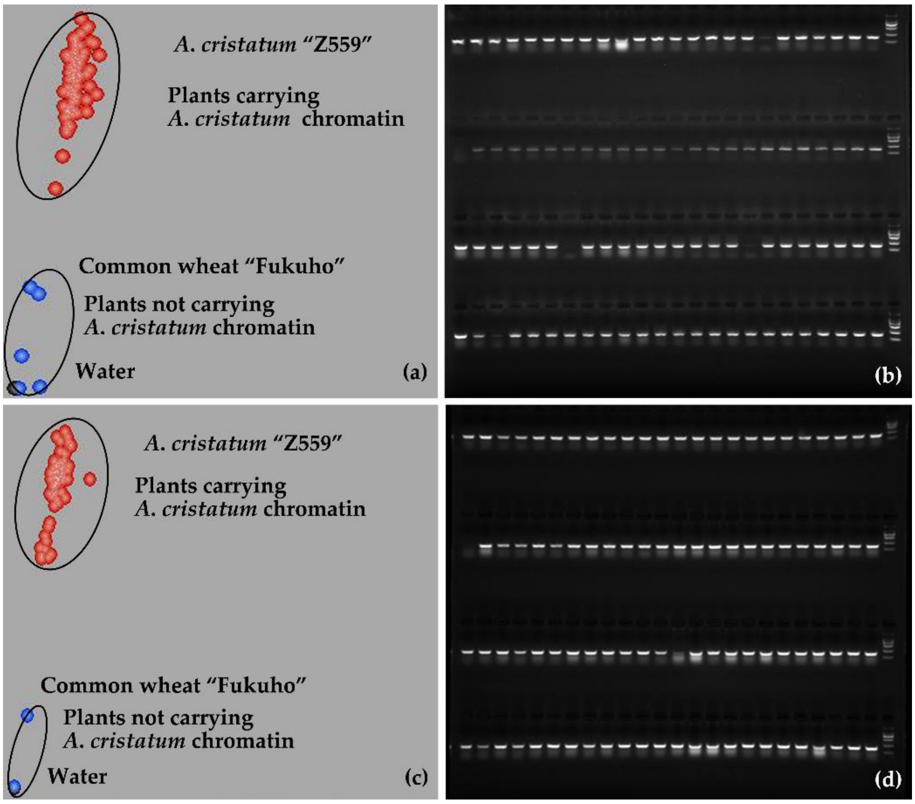


**Figure S6.** Comparison of the results of the KASP markers and the gene markers relying on agarose gel electrophoresis in the $BC_5F_{2:3}$ population of the translocation line WAT655. (a) and (b) indicated the results for the KASP marker Agr4080kps-2 and the gene marker Agr4080-3, respectively; (c) and (d) indicated the results for the KASP marker Agr6971kps-4 and the gene marker Agr6971-3, respectively. The genotype of wheat was in blue; the genotype of *A. cristatum* "Z559" was in red.

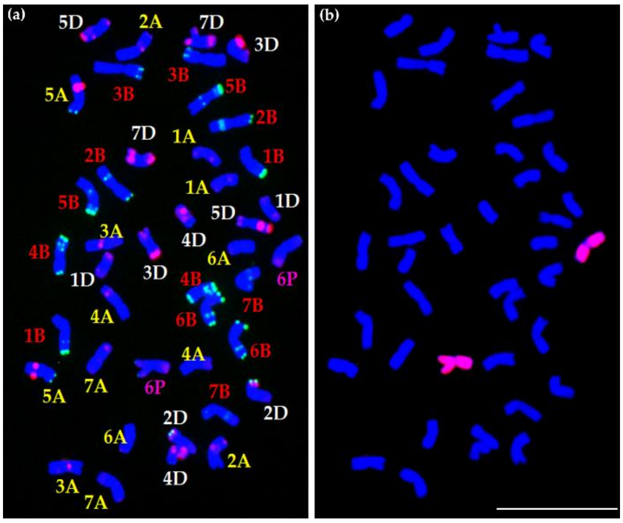


**Figure S7.** FISH and GISH patterns of the wheat-*A. cristatum* 6P disomic substitution line 4844-12-1. (a) The Oligo-pTa535-1 was in red and the Oligo-pSc119.2-1 was in green, while wheat chromosomes were in blue stained by DAPI. (b) *A. cristatum* 6P chromosomes were in red, while wheat chromosomes were in blue stained by DAPI.

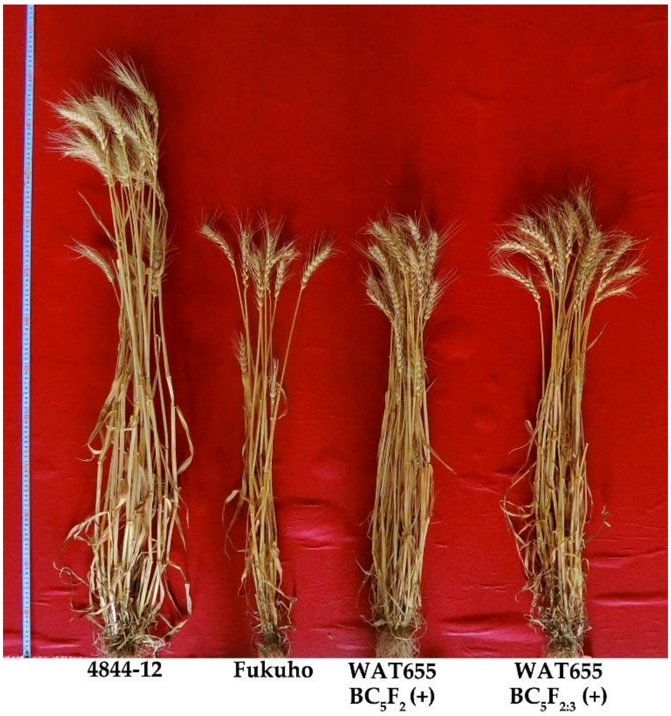


**Figure S8.** The agronomic traits of wheat-*A. cristatum* 6P disomic addition line 4844-12, common wheat "Fukuho", and wheat-*A. cristatum* 6P translocation line WAT655 ($BC_5F_2$ and $BC_5F_{2:3}$).

**Table S1.** Sequences of *A. cristatum* P genome-specific markers and the oligo probes.

| Markers | Left primer (5'–3') | Right primer (5'–3') | Annealing temperature (°C) | Reference |
|---|---|---|---|---|
| Oligo-pSc119.2-1 | 6-FAM-5′CCGTTTTGTGGACTATTACTCACCGCTTTGGGGTC CCATAGCTAT3′ | | | Tang *et al*. [58] |
| Oligo-pTa535-1 | Tamra-5′AAAAACTTGACGCACGTCACGTACAAATTGGACAAACTCTTTCGGAGTATCAGGGTTTC3′ | | | |
| AcPR2a | CTCTTTTGTTTTTGCTTTTG | CTTGGAAATCTTCTTTGTTG | 52 | Han *et al*. [45] |
| AcPR2b | GCGGCAAGGGAAAAGTAGTC | GGCATTGACCTCAGGGAAGT | 59 | |
| AcPR6 | TGAGATGACGATGGATGAAG | ACATCCTGACATTTCTGGGC | 56 | |

**Table S2.** Sequences of the KASP markers of the disease resistance-related genes.

| Markers | Left primer (5'–3') | Right primer (5'–3') |
|---|---|---|
| Agr4080kps-2 | GAAGGTCGGAGTCAACGGATTCCTTAGAGGCATAGGTTAGGTGGTAGT | GGCTACGCTGCTCGCTTCAAA |
| Agr4080kps-4 | GAAGGTCGGAGTCAACGGATTCATGATGGGGAAGGTGATGAACT | GCTAGTGACGAGGCTACGCTGCT |
| Agr6971kps-4 | GAAGGTCGGAGTCAACGGATTGATGGAATCCAGGTGGAGTTGTTT | GTAATCAATCCACAGGTTGCGAGTT |
| Agr8173kps-2 | GAAGGTCGGAGTCAACGGATTGCCGAACCACAAACGAGAGAAGT | CTGCAAACAGTCCCCACTCACC |

PCR program: an initial denaturation at 94 °C for 3 min, followed by 6 cycles of touchdown (94 °C for 20 s, touchdown at 56 °C initially and decreasing by 1 °C per cycle for 2 min), and 32–36 (for gel-based size separation) or 39 (for gel-free fluorescence signals) additional cycles of denaturation at 94 °C for 20 s, annealing/extension at 62 °C for 1 min, and a final extension at 62 °C for 2 min.
